# Next generation pan-cancer blood proteome profiling using proximity extension assay

**DOI:** 10.1038/s41467-023-39765-y

**Published:** 2023-07-18

**Authors:** María Bueno Álvez, Fredrik Edfors, Kalle von Feilitzen, Martin Zwahlen, Adil Mardinoglu, Per-Henrik Edqvist, Tobias Sjöblom, Emma Lundin, Natallia Rameika, Gunilla Enblad, Henrik Lindman, Martin Höglund, Göran Hesselager, Karin Stålberg, Malin Enblad, Oscar E. Simonson, Michael Häggman, Tomas Axelsson, Mikael Åberg, Jessica Nordlund, Wen Zhong, Max Karlsson, Ulf Gyllensten, Fredrik Ponten, Linn Fagerberg, Mathias Uhlén

**Affiliations:** 1 Science for Life Laboratory, Department of Protein Science, KTH Royal Institute of Technology, Stockholm, Sweden; 2 Centre for Host-Microbiome Interactions, Faculty of Dentistry, Oral & Craniofacial Sciences, King’s College London, London, SE1 9RT UK; 3 Department of Immunology, Genetics and Pathology, Uppsala University, Uppsala, Sweden; 4 Department of Medical Sciences, Uppsala University, Uppsala, Sweden; 5 Department of Women’s and Children’s Health, Uppsala University, Uppsala, Sweden; 6 Department of Surgical Sciences, Uppsala University, Uppsala, Sweden; 7 Department of Medical Sciences, Clinical Chemistry and SciLifeLab Affinity Proteomics, Uppsala University, Uppsala, Sweden; 8 Science for Life Laboratory, Department of Biomedical and Clinical Sciences (BKV), Linköping University, Linköping, Sweden; 9 Department of Neuroscience, Karolinska Institutet, Stockholm, Sweden

**Keywords:** Diagnostic markers, Proteomic analysis, Machine learning, Cancer genomics, Tumour biomarkers

## Abstract

A comprehensive characterization of blood proteome profiles in cancer patients can contribute to a better understanding of the disease etiology, resulting in earlier diagnosis, risk stratification and better monitoring of the different cancer subtypes. Here, we describe the use of next generation protein profiling to explore the proteome signature in blood across patients representing many of the major cancer types. Plasma profiles of 1463 proteins from more than 1400 cancer patients are measured in minute amounts of blood collected at the time of diagnosis and before treatment. An open access Disease Blood Atlas resource allows the exploration of the individual protein profiles in blood collected from the individual cancer patients. We also present studies in which classification models based on machine learning have been used for the identification of a set of proteins associated with each of the analyzed cancers. The implication for cancer precision medicine of next generation plasma profiling is discussed.

## Abstract (web summary)

Comprehensive and scalable proteomic profiling of plasma samples can improve the screening and diagnosis of cancer patients. Here, the authors use the Olink Proximity Extension Assay technology to characterise the plasma proteomes of 1477 patients across twelve cancer types, and use machine learning to obtain a protein panel for cancer classification.

## Introduction

Cancer is a highly heterogeneous disease in need of accurate and non-invasive diagnostic tools. Cancer Precision Medicine aims to enable high-resolution individualized diagnosis by the use of molecular tools such as genomics, proteomics and metabolomics, with subsequent optimized treatment and monitoring of cancer patients. Of particular importance is the possibility to identify cancers early, allowing initiation of treatment and thereby improving patient outcome by avoiding tumor progression, metastasis, and emergence of treatment resistant tumors. When cancers are detected at an earlier stage, treatment is more effective and survival is drastically improved^1^. As an example, according to US-based statistics^2^, the five-year survival for breast cancer is 99% when detected at an early stage (localized), whereas survival decreases to only 30% when detected at later stages (metastasized). Similarly, the corresponding survival for ovarian cancer is 93% at early stage and 31% when detected at later stage^2^. Based on this, several population screening programs have been initiated to identify cancer before symptoms arise, including screening for prostate cancer using PSA protein level^3^, colorectal cancer by detecting blood in feces^4^, and breast cancer using mammography^5^.

The main focus of Cancer Precision Medicine in the past decade has been to use genomics, involving next-generation sequencing to explore the genetic make-up of individual cancers. Huge efforts have been made to gain genetic insight into tumors from patients, including The Cancer Genome Atlas (TCGA)^6,7^; the International Cancer Genome Consortium (ICGC)^8^; and the Pan-Cancer Analysis of Whole Genomes (PCAWG) consortium^9^. Although invaluable insights regarding the biology of individual cancers have been gained by these efforts, the genomics information has not led to substantial changes in therapeutic regimes or facilitated screening for cancer in the population. Therefore, a move towards a multi-omics analysis has been suggested^10^, including functional analysis and alternative assay platforms, such as proteomics using either dissected tumor biopsies or non-invasive body fluids^11^.

An interesting approach in Cancer Precision Medicine is thus to use protein profiling to allow for liquid biopsy assays from minute amounts of blood. An attractive vision would be to allow multiple cancer types to be screened and detected using a single multiplex protein assay. However, the staggering dynamic range in concentrations of blood proteins spanning at least ten orders of magnitude, with concentrations as low as pg/ml for cytokines, makes multiplex analysis involving even a handful of protein targets difficult. This has hampered the development of multiplex blood protein assays during the last few decades. This situation has now changed with the recent development of high-throughput platforms for sensitive proteomics assays in blood, such as Somascan^12^ and Proximity Extension Assay (PEA)^13^. These platforms allow thousands of target proteins to be analyzed simultaneously using a few microliters of blood with sensitivity to detect and quantify proteins present in low femtomolar amounts. This means that even proteins well below the detection level for mass spectrometry can now be accurately quantified and used for population screening.

Here, we describe a strategy for pan-cancer analysis in which the plasma profiles of patients with different types of cancer are compared to find cancer-specific signatures that can distinguish each type of cancer from other cancer types. Next Generation Blood Profiling^14^, combining the antibody-based PEA with next-generation sequencing, has been used to quantify protein concentrations in multiple cancer types. Samples of more than 1400 cancer patients from a standardized biobank collection have been analyzed, along with a wealth of clinical metadata^15^. Altogether 12 cancer types including the most prevalent types such as colorectal-, breast-, lung-, and prostate-cancer, have been studied. The data is presented in the Disease Blood Atlas resource, which is available without restrictions (open access) to allow researchers both from academia and industry to explore the individual blood protein profiles from cancer patients. We also present initial studies in which classification models based on machine learning have been used to identify a panel of proteins associated with each of the analyzed cancers.

## Results

### The pan-cancer cohort

**Fig. 1:**
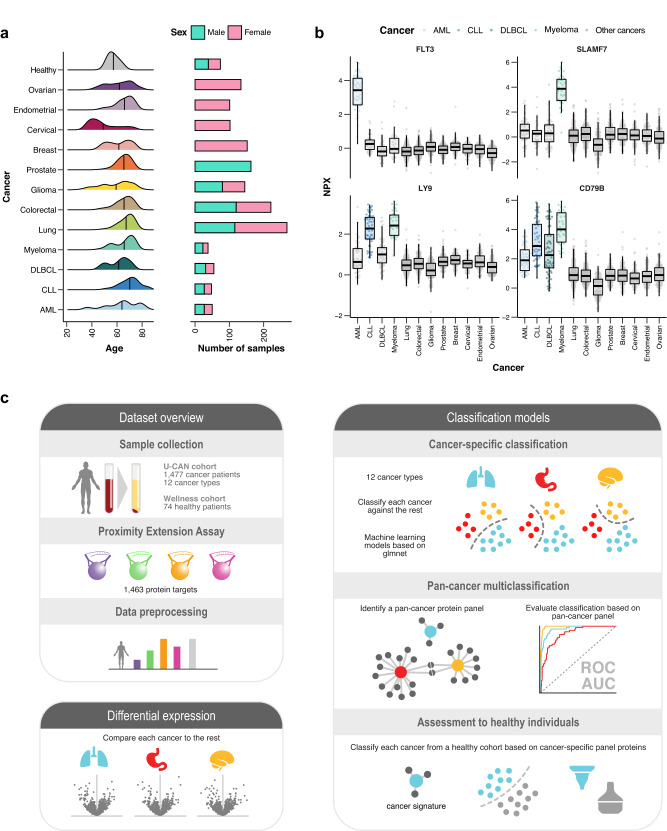
Overview of the pan-cancer study. **a** Age distribution and number of patients included for each cancer and the healthy cohort. **b** Examples of protein levels for four example proteins across the 12 cancer types. Boxplots summarize the median value, upper and lower hinges corresponding to the first and third quartiles, and whiskers indicating the minimum and maximum values within 1.5 times the IQR. Individual data points are presented for each cancer group, with *n* = 1462, *n* = 1402, *n* = 1462, and *n* = 1399 independent samples for CD79B, FLT3, LY9, and SLAMF7, respectively. **c** Schematic representation of the workflow used in this study. Blood plasma from 1477 cancer patients and 74 healthy individuals was analyzed using Proximity Extension Assay. Differential expression analysis and classification models was used to compare one cancer to all other cancers and identify cancer-associated proteins. The models for cancer classification were generated using machine learning techniques (70% of the data in training set). The resulting pan-cancer protein panel was used in a pan-cancer multiclassification strategy, and the performance tested against a test set (30% of the data) and ultimately compared against healthy individuals. Source data are provided as a Source data file. AML acute myeloid leukemia, CLL chronic lymphocytic leukemia, DLBCL diffuse large B-cell lymphoma.

In this study, we have characterized the plasma proteome of a pan-cancer cohort from the Uppsala-Umeå Comprehensive Cancer Consortium (U-CAN) biobank^15^, comprising 1477 patients from twelve cancer types, including acute myeloid leukemia (AML) (*n* = 50), chronic lymphocytic leukemia (CLL) (*n* = 48), diffuse large B-cell lymphoma (DLBCL) (*n* = 55), myeloma (*n* = 38), colorectal cancer (*n* = 221), lung cancer (*n* = 268), glioma (*n* = 145), breast cancer (*n* = 152), cervical cancer (*n* = 102), endometrial cancer (*n* = 101), ovarian cancer (*n* = 134), and prostate cancer (*n* = 163). Plasma samples were collected at the time of diagnosis and before treatment was initiated. Summary statistics for the cancer cohorts regarding age, sex, grade, and stage distribution are available in Suppl. data 1. A summary of the age distribution of the cancer patients is shown in Fig. 1a and the clinical metadata regarding age, sex, diagnosis, and cancer stage or grade available for the cancer samples are available in Suppl. data 2.

### The open access Human Disease Blood Atlas resource

The Human Disease Blood Atlas resource has been created as part of the Human Protein Atlas (v22.proteinatlas.org). This section contains more than 2 million data points representing the individual blood level for target proteins in 1477 cancer patients. The individual protein levels in blood are presented across these cancer patients characterized using the Olink Explore 1536 Proximity Extension Assay (PEA) technology, allowing the quantification of 1463 proteins using less than 3 microliters of plasma^13^. The Olink Explore has been shown to be a robust platform^13^, and we here report on the coefficient of variation (CV) with an average IntraCV of 13.3% and average InterCV of 21.1% (Fig. S1a), and a high interpanel correlation for assays used as technical controls (*r* = 0.97 for IL6, *r* = 0.96 for CXCL8 and *r* = 0.91 for TNF) (Fig. S1b). Several upregulated and downregulated proteins in specific cancer types can be observed as exemplified in Fig. 1b. Some of these potential biomarkers are cancer-specific, such as Fms-related receptor tyrosine kinase 3 (FLT3) in AML and SLAM family member 7 (SLAMF7) in myeloma, while others are found to be elevated in two or more cancers, such as lymphocyte antigen 9 (LY9) with higher expression in both CLL and myeloma. Interestingly, the B lymphocyte antigen receptor CD79b molecule (CD79B) exhibits elevated plasma levels in all four immune cell-related cancers. Figure 1c shows an overview of our workflow used to identify cancer-associated proteins based on both differential expression analysis and classification models.

### Identification of cancer-specific proteins using differential expression

**Fig. 2:**
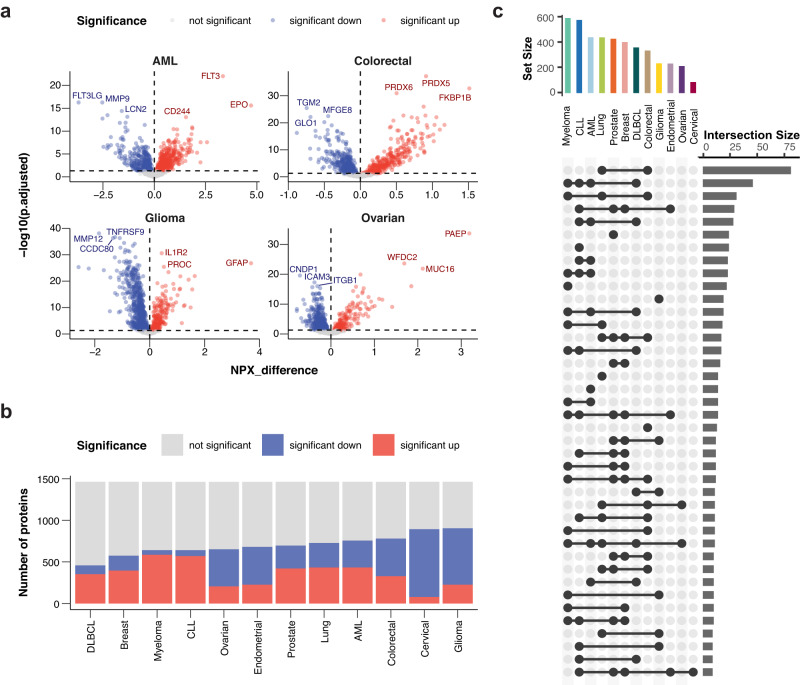
Differential expression analysis. **a** Volcano plots summarizing the differential expression results for AML, colorectal, glioma, and ovarian cancer. Corresponding results for all 12 cancers are shown in Fig. S2. *P*-values are calculated using a two-sided *t*-test, with Benjamini-Hochberg multiple hypothesis correction. **b** Barplot showing the number of proteins significantly upregulated, significantly downregulated, or with no significant differential expression for all cancer types. **c** Upset plot showing the number of upregulated proteins shared by the different cancer types. The top barplot shows the total number of upregulated proteins per cancer. Source data are provided as a Source data file. AML acute myeloid leukemia, CLL chronic lymphocytic leukemia, DLBCL diffuse large B-cell lymphoma.

To investigate the cancer-specific proteome profiles, differential expression analyses were performed where each cancer was compared to all other cancers (Fig. 1c). For the male and female cancers, only samples with the same sex were compared. The up- and downregulated proteins in each cancer are summarized by volcano plots (Fig. 2a and Fig. S2a). For glioma, the significantly upregulated proteins include the glial fibrillary acidic protein (GFAP), a protein with enriched expression in astrocytes according to the Human Protein Atlas (v22.proteinatlas.org) and for AML, the most significant protein is FLT3, a protein with elevated expression in lymphoid tissues. FKBP prolyl isomerase 1B, a protein shown by HPA to be elevated in regulatory T-cells, is upregulated in colorectal cancer, while progesterone associated emndometrial protein (PAEP), a protein secreted in the female reproductive tissues according to HPA, is significantly upregulated in ovarian cancer. The results for all 12 cancer types can be found on the interactive Disease Blood Atlas resource with links to the underlying blood levels for all analyzed proteins.

In Fig. 2b, the number of up- and downregulated proteins are shown across the 12 cancers. The results show that a large fraction of the analyzed proteins is differentially expressed. The overlap between proteins upregulated in more than one different cancer type is shown in Fig. 2c. As expected, there is a large number of upregulated proteins shared by the four immune cell-related cancers (AML, CLL, lymphoma, and myeloma), in many cases consisting of proteins related to immune-related functions. However, the largest number of overlapping proteins is observed for lung and colorectal cancer. This observation might reflect common features between these two cancer types, such as adenocarcinoma origin and a high fraction of high-grade tumors with likely similar host inflammatory response. A functional gene ontology (GO) analysis was also performed for the upregulated proteins for each of the cancer types (Fig. S2b). As expected, the upregulated proteins in the immune cell-related cancers (AML, CLL, and lymphoma) are related to immune processes, while breast, endometrial, and prostate cancer have an over-representation of cell adhesion proteins and both lung and colorectal cancer had an over-representation of apoptotic-related proteins.

### Cancer-specific classification models

To identify proteins relevant for each cancer type, a disease classification model was built for each cancer, respectively, using all measured proteins as input (*n* = 1463) and 70% of the cancer patients as the training set (Fig. 1c). To build the models, the machine learning algorithm glmnet^16^, which is based on regularized generalized linear models, was selected. The control group in each model was composed of all the other cancer samples and was subsampled to include a similar number of patients to the modeled cancer. For the male and female cancers, only samples with the same sex were used as controls.

**Fig. 3:**
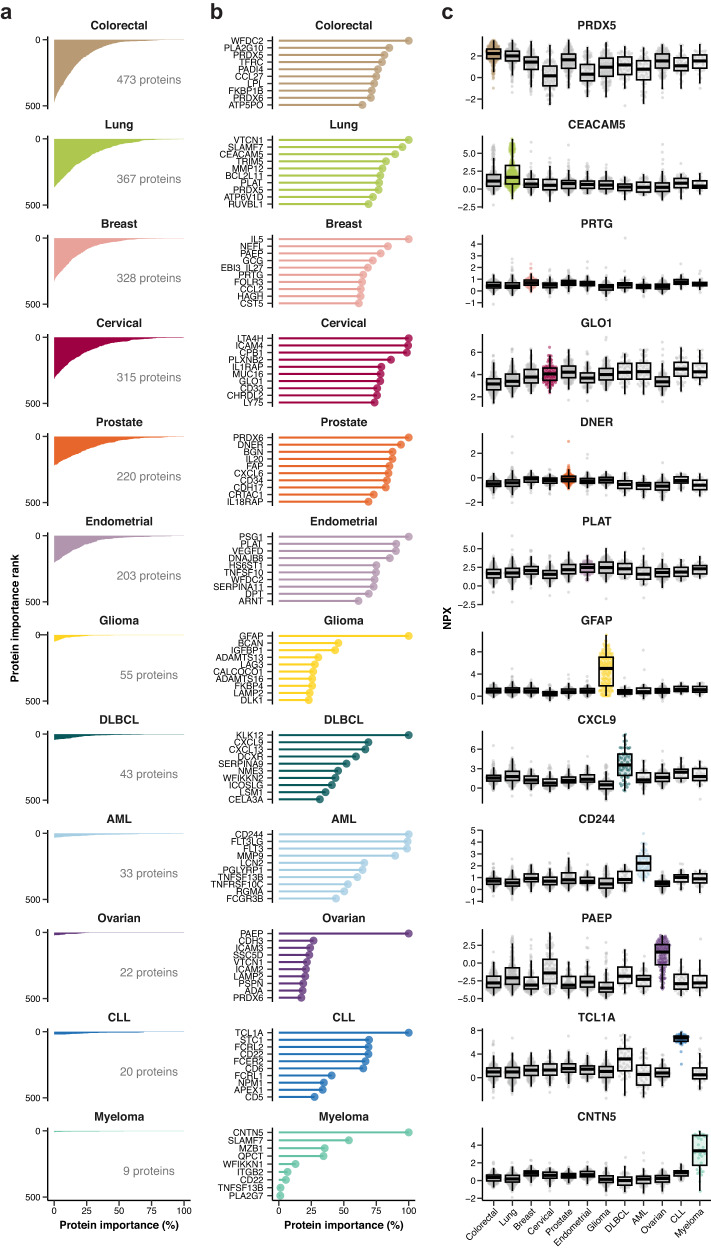
Estimation of protein importance by the cancer classification models. **a** Protein importance rank profiles for each cancer model. For each cancer, the first 500 proteins in the importance rank are included (*y*-axis), and the corresponding importance score is shown (*x*-axis). The total number of proteins with a positive score is indicated for each of the cancers. **b** Lollipop chart showing the top ten scoring proteins in each cancer model, with the exception of myeloma with only nine positive proteins. **c** Selected examples of upregulated proteins for each of the cancer types. The colored boxes indicate the cancer type where the protein is upregulated, and gray shading indicates the absence of upregulation. Boxplots summarize the median value, upper and lower hinges corresponding to the first and third quartiles, and whiskers indicating the minimum and maximum values within 1.5 times the IQR. Individual data points are presented for each cancer group, with *n* = 1462, *n* = 1402, *n* = 1457, *n* = 1413, *n* = 1432, *n* = 1476, *n* = 1402, *n* = 1432, *n* = 1462, *n* = 1389, *n* = 1389, and *n* = 1477, for PRDX5, CEACAM5, PRTG, GLO1, DNER, PLAT, GFAP, CXCL9, CD244, PAEP, TCL1A, and CNTN5, respectively. Source data are provided as a Source data file. AML acute myeloid leukemia, CLL chronic lymphocytic leukemia, DLBCL diffuse large B-cell lymphoma.

The training of a glmnet model results in an estimation of the overall importance of each protein to a model (ranging between 0–100%), revealing how many proteins are relevant to the specific classification problem and to which extent. In Fig. 3a, the number of proteins contributing to each cancer classification model is shown. Note that many proteins have a relatively high importance score for some of the cancers, including colorectal and lung cancers, while for other cancers, such as the hematological cancers and glioma, relatively few proteins contribute to the classification model. This suggests that some of the cancers require a higher number of proteins to be included in the model to classify the cancer samples from the controls. For some cancers, such as glioma, one protein (GFAP) is given a high score with considerably lower scores for the other proteins (<50%), while in other cancers there is a continuum of importance scores, such as AML or colorectal cancer. In Fig. S3a, a heatmap visualization shows the importance score for the 486 proteins that scored high (>25% importance) in at least one of the cancer types by glmnet. Moreover, several proteins scored high (>25% importance) in more than one cancer, as shown in the network visualization revealing relationships between the potential biomarkers in the different cancer types (Fig. S3b). In Fig. 3b, the ten proteins with the highest important score using the glmnet algorithm are shown for each cancer, with examples of boxplots of upregulated proteins for each cancer in Fig. 3c. The importance scores for each protein across the 12 cancer types are found in Suppl. data 3.

### Evaluation of cancer-specific classification models

**Fig. 4:**
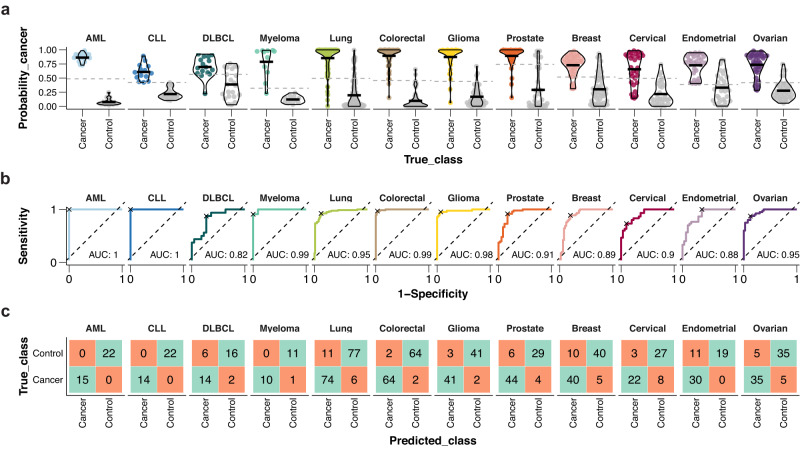
Performance of the classification models for each cancer on the test set. **a** Cancer probabilities for samples in the test set per cancer. The optimal probability cutoffs are indicated with a dashed gray line. **b** ROC curves and corresponding AUC. The sensitivity and specificity corresponding to the optimal probability cutoff is marked with an x. **c** Confusion matrices summarizing the classification results for each cancer at the given probability cutoff. The optimal probability cutoff was calculated using the Youden method. Source data are provided as a Source data file. AML acute myeloid leukemia, CLL chronic lymphocytic leukemia, DLBCL diffuse large B-cell lymphoma.

The performance of the cancer classification models was subsequently evaluated using the 30% of the data excluded from the model training. In Fig. 4a, the classification probabilities for each of the cancer models are summarized. For each cancer model, we show the probability of the plasma sample in the test set to come from the specific cancer type. We found that the machine learning models can separate samples between all the specific cancers with area under the curve AUC^16^ ranging between 0.8 and 1 (Fig. 4b). Particularly high confidence was observed for three of the immune cell-related cancers: AML, CLL, and myeloma, all having AUC of 0.99–1. To investigate the sensitivity and specificity further, a confusion matrix^17^ was created based on the probabilities estimated on the test set (Fig. 4c), with a probability cutoff calculated according to the Youden method^18^. The results suggest relatively high specificity and sensitivity across all cancers, with largest number of false positves for lung, endometrial, and breast cancers. However, the low sample size in general in the test set reinforces the need to validate the classification models in larger cohorts in the future.

In this analysis, all proteins were used as input to the model to classify the cancer types. However, to investigate the impact of using less proteins, we analyzed the classification power using different numbers of proteins as input data to the model. In Fig. S4a, the receiver operating characteristic (ROC) plots for each cancer using all proteins as input (*n* = 1463) were compared with using only the most important proteins for each cancer, including 3, 10, 50, and 200 proteins. The AUC and accuracy for each of the 12 cancers differs quite significantly as summarized in the radar plots (Fig. S4b, c) demostarting much higher AUC when using 50 or more proteins as input to the classification models for most of the cancers, although some cancers, such as AML, myeloma, and glioma, only need a few proteins to obtain high AUC scores. Additional performance scores are available in Suppl. data 4. In conclusion, this demonstrates the value of including many proteins in the classification model to gain higher confidence for some of the cancers.

### Selection of a panel with cancer-specific proteins

**Fig. 5:**
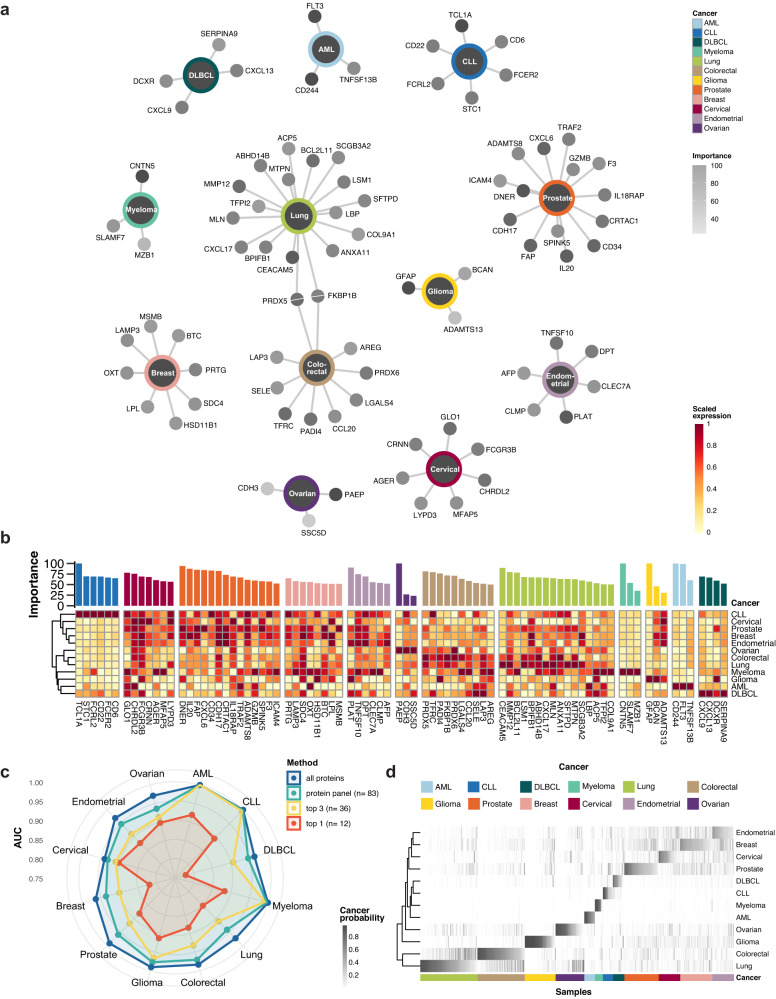
Pan-cancer protein panel and multiclassification of the pan-cancer test cohort. **a** Nework visualization of proteins included in the panel. Protein nodes are colored according to the importance score in the specific cancer. **b** Summarized expression profiles of panel proteins across the cancer types. For each protein, the scaled expression is calculated as the average NPX per cancer which is rescaled between 0 and 1. **c** Summary of the AUC for the different cancers based on models run with four different protein selections. “Top 1” and “top 3” refers to the one or three proteins with the highest importance scores for each of the individual 12 cancers models ran in the previous step, respectively, resulting in sets of 12 and 36 proteins as input to the multiclassification model. **d** Cancer probabilities for samples in the test set in the pan-cancer classification model using the panel of 83 proteins. Source data are provided as a Source data file. AML acute myeloid leukemia, CLL chronic lymphocytic leukemia, DLBCL diffuse large B-cell lymphoma.

Combining the previous results, we sought to identify a panel of proteins based on the ranking from the glmnet models and relevant to each of the analyzed cancers. The following inclusion criteria were used: (i) proteins with more than 50% overall importance as indicated by the cancer classification models, (ii) proteins identified as upregulated by differential expression analysis, and (iii) at least three proteins per cancer, which for three cancers (glioma, myeloma, and ovarian cancer) resulted in the inclusion of one or two proteins below the 50% cutoff, respectively. Based on these criteria, we ended up with a panel of 83 proteins (Fig. 5a), which are listed in Suppl. data 5 along with the results from the classification models and differential expression. Lung- and prostate cancer contributed to the largest number of proteins in the panel, 18 and 14, respectively, whereas only three protein targets each were selected for AML, glioma, myeloma, and ovarian cancer.

In Fig. 5b, the average plasma levels of the 83 selected protein members of the panel are visualized across all cancer types. Most of the selected proteins had a higher level in only one cancer, while some had high protein levels in multiple cancers. For example, CXADR-like membrane protein (CLM), selected to identify endometrial cancer, also showed elevated plasma levels in myeloma patients. Only two of the proteins were given a high importance score (> 50%) by the classification model in more than one cancer. Both FKB prolyl isomerase 1B (FKBP1B) and peroxiredoxin 5 (PRDX5) had higher plasma levels in lung- and colorectal cancer as compared to all the other cancers and were also selected independently by the models for both of these cancer types. Interestingly, FKBP1B is involved in immunoregulation and protein folding and has previously been linked to colorectal cancer^19^ but not to lung cancer. Similarly, PRDX5 has an antioxidant function in normal and inflammatory conditions and several other proteins of the peroxiredoxin family have been linked to lung and colorectal cancers in transcriptomics analysis of cancer cell lines^20,21^.

### Classification of the pan-cancer cohort based on the selected protein panel

Next, we aimed to assess whether a multiclass classification model based on the selected protein panel could result in an accurate classification of samples of the different cancer types. Here, a glmnet model was built using all previous cancer samples from the training set and the performance was estimated on all cancer samples on the test set, looking at the ability of the model to score each sample with a probability to belong to each of the cancer types. In order to explore the impact of including different number of proteins, we built four different multiclass classification models based on a different selection of proteins: (i) all proteins (*n* = 1463), (ii) those selected in the panel (*n* = 83), (iii) the three most important proteins per cancer (*n* = 36) and (iv) the single most important protein per cancer (*n* = 12), and we evaluated the performance in each setting. Comparative ROC analyses were performed for each cancer type in which the specificity/sensitivity measured as AUC was determined for different number of proteins (Fig. S5).

The results (Fig. 5c) show that the panel of 83 proteins can identify the right cancer with relatively high selectivity and sensitivity with AUC ranging between 0.93 and 1 for all cancer types. The analysis using all proteins gave only slightly better results, while the use of only the top 3 proteins in each cancer gave somewhat less reliable results. The lowest performance scores were obtained when using only the top protein for each of the 12 cancers. Additional performance scores for the different protein numbers are summarized for each of the cancers in Suppl. data 6.

The results demonstrate that a panel with only a small number of protein markers can achieve similar classification reliability as using all proteins. Although based on a small sample size in the test cohort, the results suggest that a panel of less than hundred proteins yields highly promising results (AUC) for simultaneous identification of all 12 cancer types. As shown in Fig. 5d, there is some overlap in the classification results for some of the cancers, such as lung and colorectal cancer, while for other cancers, such as glioma and immune-related cancers, the samples have a high probability of being correctly classified.

### Comparative analyses between healthy individuals and patients with cancer

An important question is how well the protein signature identified on the pan-cancer study can distinguish cancer patients from healthy individuals. To investigate this, for each of the 12 cancer types, a cancer classification model was built but this time including 74 healthy individuals previously studied as part of a wellness study^14,22,23^ as the control group instead of all of the other cancers. As described above, each of the cancers contributed to the panel with a different number of proteins^3–18^ and these models were based only on these specific proteins, i.e., the AML model was based on the three AML-specific proteins included in the panel. We again used 70% of the cancer and healthy samples as the training set and the remaining 30% to test the performance of the model, being the cancer samples in the train and test set the same as before.

**Fig. 6:**
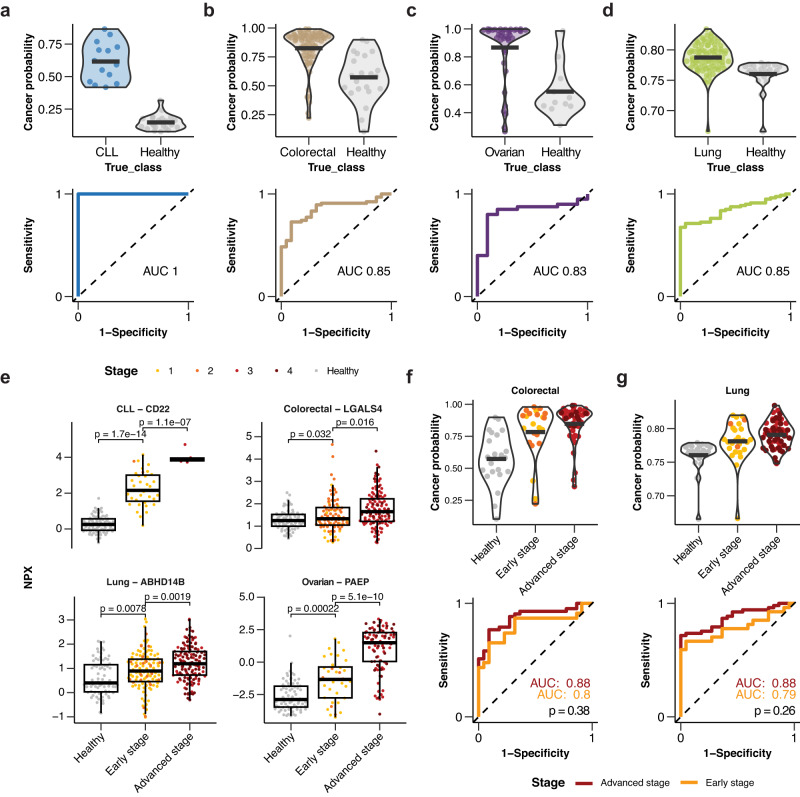
Classification of cancer samples against a healthy cohort based on the selected protein panel. Model results showing the cancer probability for cancer and healthy individuals from the test set (top) and the ROC curve with AUC score (bottom) for **a** CLL, **b** colorectal cancer, **c** ovarian cancer, **d** lung cancer. **e** Protein levels of four different proteins for cancer samples stratified into early (stage 1–2) or advanced (stage 3–4) stages as well as the healthy cohort. Boxplots summarize the median value, upper and lower hinges corresponding to the first and third quartiles, and whiskers indicating the minimum and maximum values within 1.5 times the IQR. Individual data points are presented for each cancer group, with *n* = 327, *n* = 114, *n* = 289, and *n* = 200, for ABHD14B, CD22, LGALS4, and PAEP, respectively. *P*-values are calculated using a two-sided t-test to compare the group means. Model results showing the cancer probability for cancer samples stratified by stage (early or advanced) and healthy individuals (top) and the ROC curve with AUC score (bottom) for **f** colorectal cancer and **g** lung cancer. The *p*-values are calculated using unpaired DeLong’s test. Source data are provided as a Source data file. CLL chronic lymphocytic leukemia.

The results for four of the cancers are shown in Fig. 6a–d and all cancers in Fig. S6. For CLL (Fig. 6a), the model can distinguish cancer patients from healthy controls using the six proteins selected for CLL with total accuracy (AUC = 1). Similarly, the same analysis for colorectal- (Fig. 6b), ovarian- (Fig. 6c), and lung cancer (Fig. 6d), respectively, shows high accuracy with all AUC results above 0.83 when using the corresponding proteins, demonstrating that the selected cancer signatures can distinguish cancer patients from healthy individuals with relatively high accuracy. Additional performance metrics are provided for all models in Suppl. data 7. These results suggest that the protein panel is suitable to classify patients with the analyzed cancer types from each other as well as distinguish cancer patients from healthy individuals (without a cancer diagnosis). However, caution is required since the wellness panel was sampled and analyzed in a separate study, thus sample bias can not be ruled out.

### Stratification of patients with cancers of different stages

An important quest in the field of Cancer Precision Medicine is to aid clinicians to indicate the stage of the cancer. For some cancers in this study, a relatively large number of patients had stage data available and therefore we investigated whether the protein panel could stratify patients into stages for these cancer types. In Fig. 6e, we show four examples of proteins where we find an association between the plasma levels and disease stage, including (i) CD22 used to identify CLL patients; (ii) galectin 4 (LGALS4) in colorectal cancer patients; (iii) arbhydrolsase domain containing 14B (ABHD14B) in lung cancer patients; and (iv) the ovarian cancer biomarker Progestagen associated endometrial protein (PAEP). These examples demonstrate the possibility to perform stage stratification simply by analyzing selected plasma protein levels, but further analyses in additional cohorts are needed to demonstrate the validity of the protein panel for cancer stage stratification.

### Classification of early-stage cancer samples

One of the most important objectives in the field of cancer precision medicine is to identify cancer at an early stage to provide successful therapeutic intervention and to improve patient survival. To assess the ability of the protein panel to distinguish early-stage cancer from healthy individuals, we stratified the ROC analysis into the early (stage 1 and 2) and advanced (stage 3 and 4) stages for colorectal and lung cancer, where we have the largest sample sizes for patients across stages (Fig. S7 and Fig. 6f, g). In Fig. 6f (top), the cancer probability score for lung cancer patients across stages is compared with the corresponding score for healthy individuals. A clear difference in score is shown for most samples and the AUC score (Fig. 6f, bottom) for separating early-stage colorectal cancer patients from healthy individuals is 0.80. Similarly, for the early-stage lung cancer patients, a clear difference in the estimated probabilities is observed between early-stage cancer and healthy samples by the protein panel model (Fig. 6g, top), and the corresponding AUC score (Fig. 6g, bottom) is 0.79. In both cases, there is no significant difference between the model performance on early and advanced stage cancer patients. This highlights the potential of the selected biomarker panel to identify early-stage colorectal and lung cancer patients, although more in depth analysis in independent cohorts is warranted.

## Discussion

Here, we describe a strategy based on next-generation plasma profiling to explore the cancer proteome signatures by comprehensively exploring the protein levels in patients representing most major cancer types. The study describes and compares the plasma proteome across all major cancers using a multiplex assay platform. The platform allows thousands of proteins to be quantitatively analyzed using only a few microliters of blood opening up new opportunities for Precision Cancer Medicine. The plasma levels of each individual protein have been determined for more than 1400 cancer patients representing 12 different cancer types, and the results for the individual protein targets are presented in the open access Human Disease Blood Atlas (v22.proteinatlas.org/humanproteome/disease).

We have used the data to identify a set of proteins associated with each of the cancers studied using machine learning. A classification model based on a restricted set of 83 upregulated proteins was built and the accuracy of the classification of pan-cancer samples was evaluated in a separate test cohort. It is interesting to observe the dramatic increase in classification performance when using the protein panel (*n* = 83) as compared to the use of only the top protein marker for each cancer. This demonstrates the added advantage of using a panel of blood proteins, as exemplified by patients with breast cancer for which individual markers are relatively unselective, but the classification model using multiple proteins gave a potentially much more accurate classification.

The panel allowed the stratification of plasma samples from most cancer types with high sensitivity and specificity and it was also able to detect patients with early disease, as exemplified by early-stage patients in lung and colorectal cancers. However, in this context it is important to point out that the test cohorts used for the various cancer validations were relatively small sized and additional validation cohorts are needed to confirm the validity of each protein in the classification model. For example, in two earlier studies of blood from glioma patients^24,25^, only a few upregulated proteins were found and none of these were significantly upregulated here. This demonstrates the importance of several independent studies before establishing a pan-cancer protein panel. The performance of the classification model and the utility of the protein panel need to be validated in independent cohorts before consideration for clinical use. Of particular importance is validation in a large background of non-diseased individuals to establish the breadth of false positives. It is also desirable to have the results validated by independent technical platforms, such as sandwich^26^, mass spectrometry^27^, or Somascan^12^ assays.

The proteins used in the classification models include well-known markers for some of the cancers, but also proteins with, to our knowledge, no previous connection to cancer. It is noteworthy that the cancer-specific elevation of the panel proteins in blood plasma could reflect several underlying causes, such as an increase of leakage or secretion from the tumor or surrounding tissue itself, or due to the bodily response to the tumor. However, a more in-depth analysis is needed to explain the causal relationship between the proteins and the respective cancer types.

As mentioned above, it is noteworthy that individual variation of protein plasma levels in both healthy and disease states calls for validation of potential biomarkers using an independent assay platform as well as using independent patient cohorts. Since even a highly selective assay used in a population screening still could generate a large number of false positives, when millions of individuals are screened for presence of cancer, it is particularly important to rule out false positives, which could cause considerable and unnecessary stress for the individual. It is thus important for any screening procedure to be followed up by independent validation, such as mammography for breast cancer, blood in feces and/or colon spectroscopy for colorectal cancer, radiological examination, and/or tissue-based analysis of biopsies for many other cancers. This makes it possible to combine initial and broad population screening with less cost-effective assay platforms to establish the diagnosis of patients with cancer.

It is of course interesting to expand the analysis presented here to add other frequent and important cancers to the pan-cancer strategy, such as liver, kidney, and pancreatic cancers. Similarly, it is also valuable to compare the cancer profiles reported here with plasma profiles from patients having other diseases. Our aim in the near future is to be able to report such studies as part of the open access Human Disease Blood Atlas resource for patients in the field of cardiovascular, autoimmune, neurological, and infectious disease, among others. It is also interesting to add more protein targets to the analysis and such larger panels are now available for exploration by both the PEA^13^ technology, which currently can analyze 3000 targets, and the Somascan platform^12^, including 7000 targets.

In summary, we describe a strategy for exploration of protein profiles in blood with the ultimate objective to allow simultaneous identification of cancers using few microliters of blood. Since the analytical platform used here can be combined with simple sample collection formats such as dried blood spots, cost-effective pan-cancer population screening can be foreseen in which a panel of proteins are used to identify multiple cancer types in a single assay. Such population screenings could be organized to allow the discovery of cancers early and thus help clinicians to start treatment of cancer patients at earlier stages. It is our hope that the data in the open access Human Blood Disease database will be a valuable resource for such future efforts in the field of Cancer Precision Medicine.

## Methods

The research complies with all relevant ethical regulations. The pan-cancer study was approved by the Swedish Ethical Review Authority (EPM dnr 2019-00222). The research was in line with donor consents in U-CAN (28631533, EPN Uppsala 2010-198 with amendments), and all participants provided written informed consent. The Wellness healthy cohort study was approved by the Ethical Review Board of Goteborg, Sweden (registration number 407-15), and all participants provided written informed consent. The study protocol conforms to the ethical guidelines of the 1975 Declaration of Helsinki.

### The pan-cancer study cohort

Plasma samples from 1477 cancer patients were obtained from the U-CAN biobank which collects samples from consenting patients diagnosed at the Akademiska hospital in Uppsala as part of the clinical routine and with a high degree of standardization^15^. Plasma samples were obtained from treatment-naïve patients taken around the time of their diagnosis. Plasma was prepared from whole blood by centrifugation at 2.400 × *g* for seven minutes at room temperature, after which the plasma was aliquoted into several 220 µl vials and immediately frozen for long-term storage at −80 °C. Exclusion criteria included any concurrent or previous cancer within the last five years, and arm-to-freezer time exceeding 360 min. Diagnosis, stage, age, sex and other variables were obtained from the U-CAN database and the patient’s clinical records.

### The Wellness healthy cohort

Plasma samples from healthy individuals (39 males and 35 females) were selected from the first sampling time point of the Swedish SciLifeLab SCAPIS Wellness Profiling (S3WP) study as described previously^22,23^. The selection process aimed to include patients with the most complete data available for all sampling time points across multiple datasets. The S3WP program includes longitudinal samples from 101 healthy individuals aged 50–64, recruited from the prospective observational Swedish CArdioPulmonary bioImage Study (SCAPIS) sampled at six different time points during a 2-year period.

### Measurement of protein levels

The protein levels of all 1477 cancer samples were measured in plasma using the Olink Explore PEA technology^13^, which uses antibody-binding capabilities to detect the levels of 1463 targets in plasma coupled with next-generation sequencing (NGS) readout. The Wellness healthy cohort had previously been analyzed in the Olink Explore as described in Zhong et al.^14^ and 16 samples from this study were included in the cancer study to allow for bridging between the results for the two cohorts. The Olink Explore 1536 platform includes four different panels: the Olink Explore 384 Cardiometabolic Reagent Kit (Panel lot number: B04413, Product number: 97700/97300), the Olink Explore 384 Inflammation Reagent Kit (Panel lot number: B04411, Product number: 97500/97100)), the Olink Explore 384 Oncology Reagent Kit (Panel lot number: B04412, Product number: 97600/97200)), and the Olink Explore 384 Neurology Reagent Kit (Panel lot number: B04414, Product number: 97800/97400). A total of 1472 proteins were targeted using specific antibodies, including 1463 unique proteins as well as controls. Each antibody was conjugated separately with two complementary probes, and distributed in four separate 384-plex panels, focused on the four disease areas: cardiovascular, inflammation, neurology, and oncology. In brief, the PEA workflow started with an overnight incubation to allow the conjugated antibodies to bind to the corresponding proteins in the samples. The incubation was followed with an extension and pre-amplification step when the hybridization and extension of complementary probes takes pace. The extended DNA was then amplified by PCR and further indexed to allow the preparation of libraries, which were then sequenced using Illumina’s NovaSeq platform. The counts obtained from the sequencing run were subjected to a quality control and normalization procedure. Here, internal controls introduced at different steps were used to reduce intra-assay variability. These include an incubation control consisting of a non-human antigen measured with the same technology, an extension control consisting of an antibody conjugated to a unique pair of probes which are in proximity and is expected to produce a positive signal, and a control in the amplification step consisting of a double-stranded DNA sequence which is expected to produce a positive signal independent of the amplification step. Additionally, external controls such as negative control (buffer sample) and plate controls (pool of plasma) were used to establish a limit of detection (LOD) and adjust levels between plates, respectively. Finally, two known samples acted as sample controls to calculate the precision of the measurements. After quality control and normalization, the data was provided in the relative protein quantification unit Normalized Protein eXpression (NPX) unit, which is on a log2 scale. The NPX score is calculated based on matched counts from the sequencing data and a high NPX value can be interpreted as a high protein level. All measurements that failed the internal quality control and thus reported with a warning were excluded from the dataset. Three of the protein assays (IL6, CXCL8, and TNF) were included in all four panels for quality assurance purposes and were used as technical controls to investigate the quality of the samples using the interpanel correlation between all NPX values above the give limit of detection range (LOD)^13^. In addition, the coefficient of variation (CV) of each assay was calculated as a measure of the technical variance within a plate (IntraCV) and across several plates (InterCV), based on the pooled plasma sample run in duplicate on each plate in the Olink Explore setup, following the procedure as presented in Wik et al.^13^.

### Differential expression analysis

The differential protein expression was assessed using a two-sided t-test coupled with Benjamini-Hochberg multiple hypothesis correction^28^, with a significance threshold of 0.05 for adjusted *p*-values. The adjusted *p*-values and difference in average expression per group were summarized in volcano plots for each of the analyzed cancers. Enrichment analysis of upregulated protein sets were performed using the clusterProfiler package (version 3.18.1)^29^. The enricher() function in clusterProfiler was used to perform overrepresentation analysis against the biological annotations from Gene Ontology (GO) biological processes (BP)^30^, with subsequent *p*-value adjustment using the Benjamini-Hochberg method^28^ and using adjusted *p*-value < 0.05 as threshold for significance.

### Disease classification models

Classification models were built in three different settings: (1) to classify patients with one cancer from patients with other cancers, (2) to classify all cancers simultaneously, and (3) to classify patients with a specific cancer from healthy samples. All models were built using the caret R package (v 6.0.90)^31^.

First, the cancer and wellness data were split in 70% for training purposes and 30% for testing purposes using the createDataPartition() function in caret, generating a training and testing pool of samples. For all models described, the test and train sets were composed of a subset of the training and testing pool sets, to avoid data leakage^32,33^. In the first setting, the training set for the classification of a specific cancer was composed of all samples from that cancer in the training pool and a balanced equally sized subset of samples from all other cancers acting as controls. In the same manner, the test set was composed of all samples from that cancer in the testing pool and matching number of controls representing all other cancers. For cancers consisting of male or female samples exclusively, only samples from the same sex were used as controls. In the multiclassification setting, all cancer samples in the training and testing pools, respectively, were combined into two large set of samples used for training and testing. Finally, when classifying patients from one cancer against the healthy cohort, all samples from that cancer and healthy patients were used, with samples in the training pool being used for training the model and samples in the testing pool being used for testing. Again, only male or female samples were used as control for male and female-specific cancers, respectively.

Before the model training, the data with missing values due to failed quality control was imputed using the preProcess() function in caret with the “knnImpute” method. Batch correction using the removeBatchEffect() function in the limma package (version 3.46.0)^34^ was performed to correct for potential batch effects between the cancer and healthy samples. The cancer prediction models were built on the selected training sets using the function train() in caret, and glmnet was used as the classification algorithm^16^. A 5-fold cross-validation scheme and built-in parameter tuning were applied to the models. The contribution of each protein to the model was retrieved using the varImp() function in the caret package. When indicated, the data used as input to the model was restricted to a subset of proteins, which was guided by the feature importance ranking obtained when training the model using all proteins and thus based solely on training data.

The predict() function in caret was used to estimate the class probabilities for the samples in the test set, which were not part of the training of any of the models and allowed an unbiased estimation of model performance. ROC analyses were performed to assess the sensitivity and specificity of the classification, summarized as AUC scores. The pROC R package (v 1.18.0) was used for binary classifications and multiROC (v 1.1.1) was used for multiclass classification. Statistical significance for differences in AUC were calculated using the DeLong test^35^ implementation in the pROC package, using *p*-value < 0.05 as the threshold for significance. Additionally, sensitivity, specificity, positive predictive value (PPV), negative predictive value (NPV), precision, recall, and F1 scores were calculated. For the binary classifications, these metrics were based on a probability threshold estimated using the coords() function in pROC with the Youden index^18^.

### Data visualization

Data visualization was performed in R (version 4.0.3)^36^, using the ggplot2 (version 3.3.5)^37^, ggbeeswarm (version 0.6.0)^38^, ggpubr (version 0.5.0)^39^, ggraph (version 2.0.5)^40^, ggrepel (version 0.9.1)^41^, ggridges (version 0.5.3)^42^, ggplotify (version 0.1.0)^43^, igraph (version 1.2.6)^44^, pheatmap (version 1.0.12)^45^, patchwork (version 1.1.1)^46^, tidygraph (version 1.2.0)^47^, and UpSetR (version 1.4.0)^48^ packages. For the heatmap visualization, data was rescaled to a 0–1 scale and hierarchical clustering was performed using the “ward.D2” method. The limma R package (version 3.46.0)^34^ was used to correct for batch differences for the comparison between the U-CAN cancer cohorts and the Wellness healthy cohorts. The figures were assembled in Affinity designer (version 1.10.0.1127).

### Reporting summary

Further information on research design is available in the Nature Portfolio Reporting Summary linked to this article.

## .Supplementary information

Supplementary Information

Description of Additional Supplementary Files

Supplementary data 1

Supplementary data 2

Supplementary data 3

Supplementary data 4

Supplementary data 5

Supplementary data 6

Supplementary data 7

Reporting Summary

## Data Availability

The normalized U-CAN proteomics data generated in this study have been deposited in the BioStudies database under accession code S-BSST935, as well as on the Human Protein Atlas data publication page [https://www.proteinatlas.org/about/publicationdata]. All proteins are also visualized on the individual protein summary pages of the Human Disease Blood Atlas. For the Wellness healthy cohort, the Olink Explore participant-level datasets have been deposited with the Swedish National Data Service [https://snd.gu.se/sv/catalogue/study/preview/88efa94d-39b3-4a50-8b3b-87b1abedefd4], and the data have been previously published^14^. Due to patient consent and confidentiality agreements, the datasets can be made available only for validation purposes by contacting snd@snd.gu.se. Data access will be evaluated according to Swedish legislation. Data access for research-related questions in the S3WP program can be made available by contacting the corresponding author. Source data are provided with this paper.

## Source data

Source Data

## Code availability

All code necessary for the data analysis and visualization is available at https://github.com/buenoalvezm/Pan-cancer-profiling^49^.
