# Supplementary material for: Next generation pan-cancer blood proteome profiling using proximity extension assay
Source: Nat Commun. 2023 Jul 18;14:4308. doi: 10.1038/s41467-023-39765-y (PMC10354027; doi:10.1038/s41467-023-39765-y)
Supplement: Supplementary file 1 — Supplementary Information [file 41467_2023_39765_MOESM1_ESM.pdf]

Supplementary Materials for

Next generation pan-cancer blood proteome profiling using proximity extension assay

María Bueno Álvez[1], Fredrik Edfors[1], Kalle von Feilitzen[1], Martin Zwahlen[1], Adil Mardinoglu[1,2], Per-Henrik Edqvist[3], Tobias Sjöblom[3], Emma Lundin[3], Natallia Rameika[3], Gunilla Enblad[3], Henrik Lindman[3], Martin Höglund[4], Göran Hesselager[4], Karin Stålberg[5], Malin Enblad[6], Oscar E. Simonson[6], Michael Häggman[6], Tomas Axelsson[4], Mikael Åberg[7], Jessica Nordlund[4], Wen Zhong[8], Max Karlsson[1], Ulf Gyllensten[3], Fredrik Ponten[3], Linn Fagerberg[1] and Mathias Uhlén[1,9*]

[1]Science for Life Laboratory, Department of Protein Science, KTH Royal Institute of Technology, Stockholm, Sweden; [2]Centre for Host-Microbiome Interactions, Faculty of Dentistry, Oral & Craniofacial Sciences, King's College London, London SE1 9RT, UK; [3]Department of Immunology, Genetics and Pathology, Uppsala University, Sweden; [4]Department of Medical Sciences, Uppsala University, Sweden; [5]Department of Women's and Children's Health, Uppsala University, Sweden; [6]Department of Surgical Sciences, Uppsala University, Sweden; [7]Department of Medical Sciences, Clinical Chemistry and SciLifeLab Affinity Proteomics, Uppsala University, Sweden; [8]Science for Life Laboratory, Department of Biomedical and Clinical Sciences (BKV), Linköping University, Linköping, Sweden and [9]Department of Neuroscience, Karolinska Institutet, Stockholm, Sweden. [*]Corresponding author

Corresponding auhor: Mathias Uhlén (mathias.uhlen@scilifelab.se)

This PDF file includes:

Figures S1 to S7

Other Supplementary Materials for this manuscript include the following:

Supplementary Data 1 to 7

Figure S1

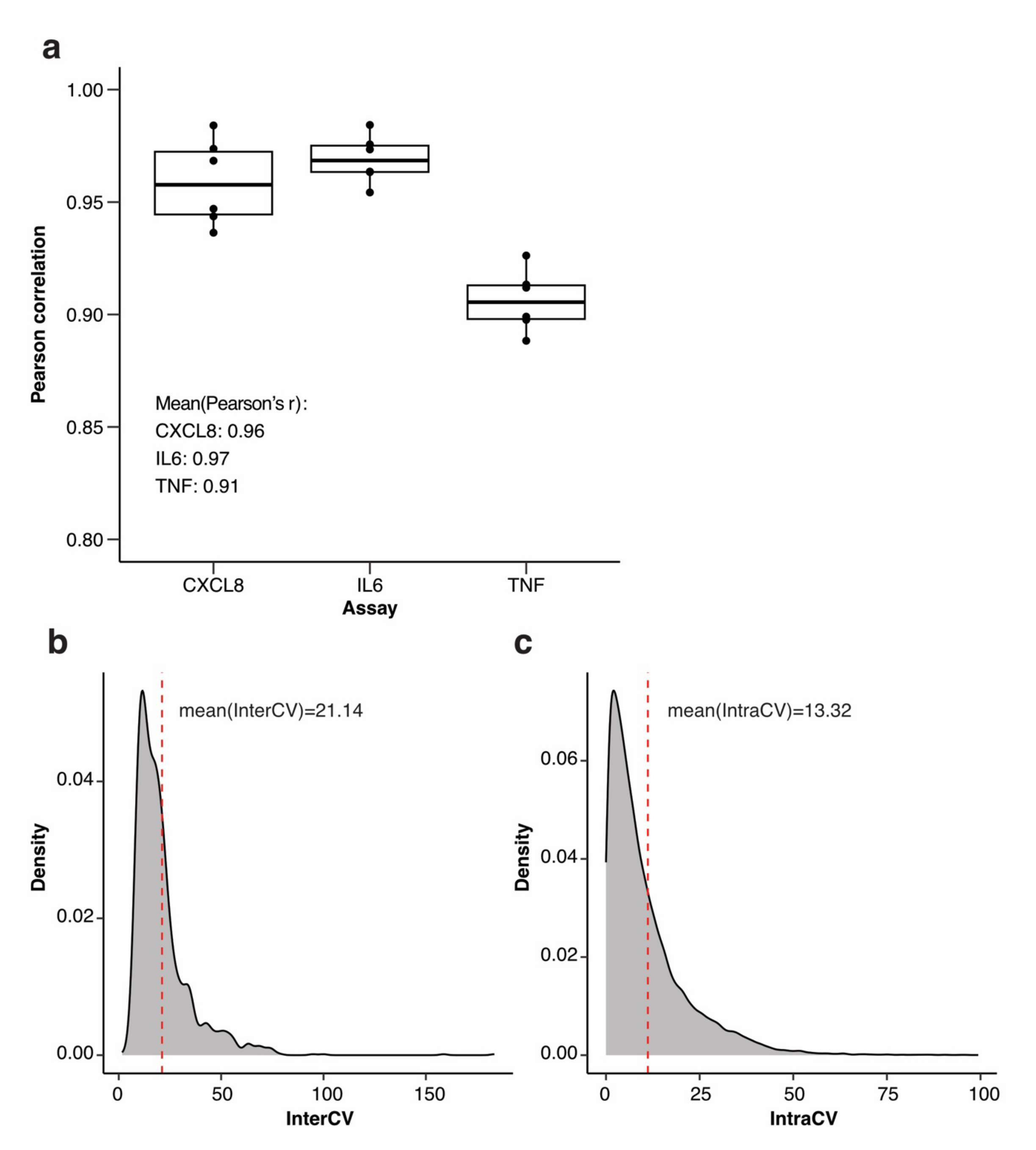

Fig S1 | Quality control of the PEA data. **a**, Boxplot showing the interpanel correlation for the three assays (CXCL8, IL6 and TNF) run in each of the four 384-plex panels and used as technical controls. Boxplots summarize the median value, upper and lower hinges corresponding to the first and third quartiles, and whiskers indicating the minimum and maximum values within 1.5 times the IQR. Individual data points are presented for each protein, with n = 6 for CXCL8, IL6 and TNF. **b**, Distribution of CV values for each assay across several plates (InterCV) based on pooled plasma control samples. Dashed red line denotes the average InterCV. **c**, Distribution of CV values for each assay within the same plate

(IntraCV) based on pooled plasma control samples. Dashed red line denotes the average IntraCV. Source data are provided as a Source Data file. CV: coefficient of variation.

Figure 2

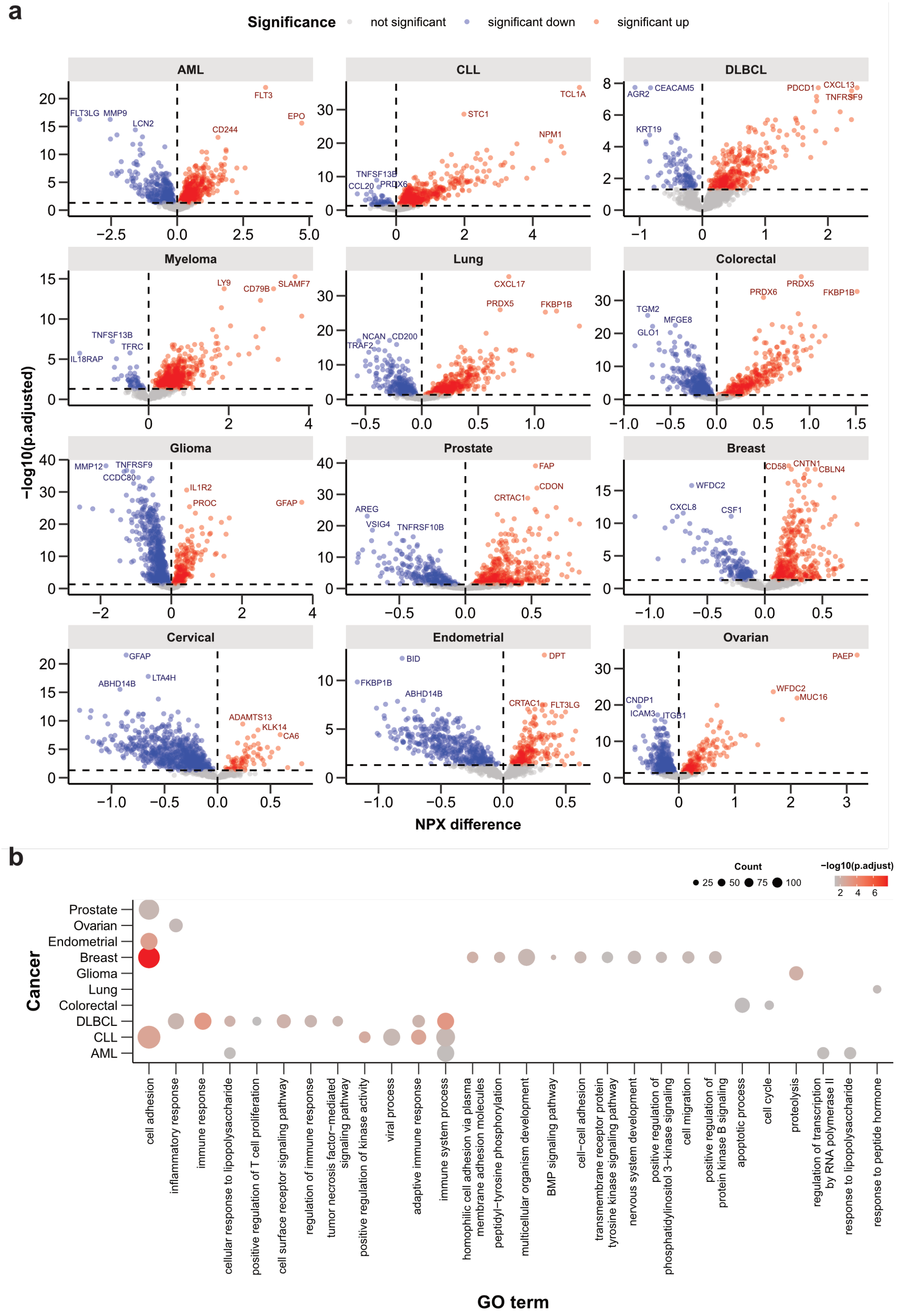
a
Significance not significant significant down significant up
AML
CLL
DLBCL
Myeloma
Lung
Colorectal
Glioma
Prostate
Breast
Cervical
Endometrial
Ovarian
−log10(p.adjusted)
NPX difference
b
Count 25 50 75 100
−log10(p.adjust) 2 4 6
Cancer
Prostate
Ovarian
Endometrial
Breast
Glioma
Lung
Colorectal
DLBCL
CLL
AML
cell adhesion
inflammatory response
immune response
cellular response to lipopolysaccharide
positive regulation of T cell proliferation
cell surface receptor signaling pathway
regulation of immune response
tumor necrosis factor−mediated signaling pathway
positive regulation of kinase activity
viral process
adaptive immune response
immune system process
homophilic cell adhesion via plasma membrane adhesion molecules
peptidyl−tyrosine phosphorylation
multicellular organism development
BMP signaling pathway
cell−cell adhesion
transmembrane receptor protein tyrosine kinase signaling pathway
nervous system development
positive regulation of phosphatidylinositol 3−kinase signaling
cell migration
positive regulation of protein kinase B signaling
apoptotic process
cell cycle
proteolysis
regulation of transcription by RNA polymerase II
response to lipopolysaccharide
response to peptide hormone
GO term

Fig. S2 | Differential expression and GO enrichment analysis for each cancer. a, Volcano plots based on differential expression analysis for each cancer against all other cancers. The y-axis corresponds to the adjusted p-value, while the x-axis shows the difference in average protein expression (NPX) in a specific cancer compared to all other cancers. P-values are calculated using a two-sided t-test, with Benjamini-Hochberg multiple hypothesis correction. **b**, GO enrichment analysis based on upregulated proteins per cancer. The plot shows Biological Processes (BP) terms enriched in each of the upregulated protein sets. The dot size is proportional to the number of genes associated to a specific GO term while the color indicates the significance of the association. P-values are calculated using a hypergeometric test, with Benjamini-Hochberg multiple hypothesis correction. Source data are provided as a Source Data file. AML: acute myeloid leukemia, CLL: chronic lymphocytic leukemia, DLBCL: diffuse large B-cell lymphoma.

Figure S3

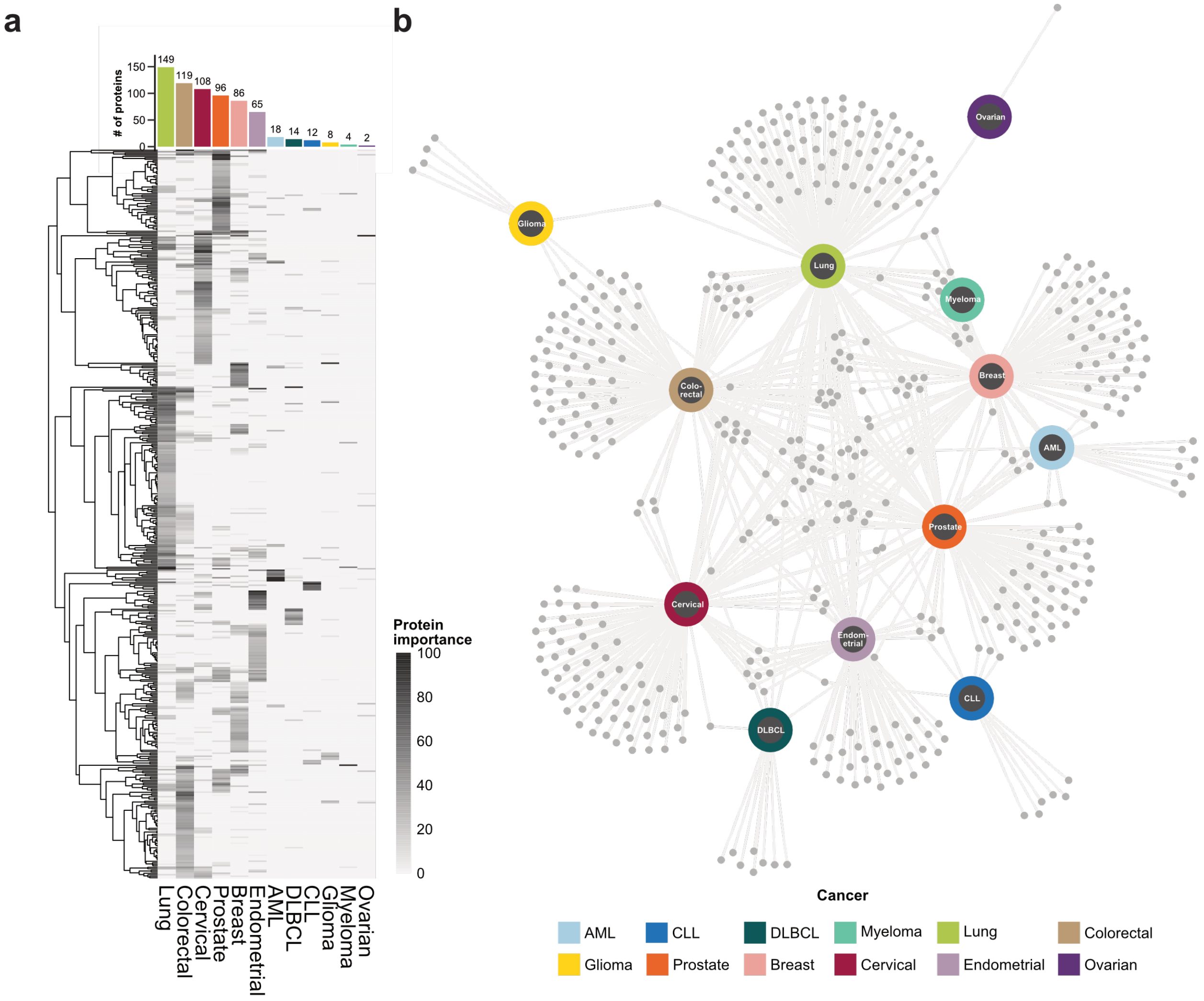

Fig S3 | Protein importance in the cancer classification models. a, Heatmap visualization of importance score across cancers for proteins with > 25% importance in one cancer (n= 486), where each row represents one protein. The barplot shows the number of proteins with >25% importance in each of the cancers. **b**, Network visualization of importance score across cancers for proteins with > 25% importance in one cancer (n= 486), where each grey dot represents a protein. Source data are provided as a Source Data file. AML: acute myeloid leukemia, CLL: chronic lymphocytic leukemia, DLBCL: diffuse large B-cell lymphoma.

Figure S4

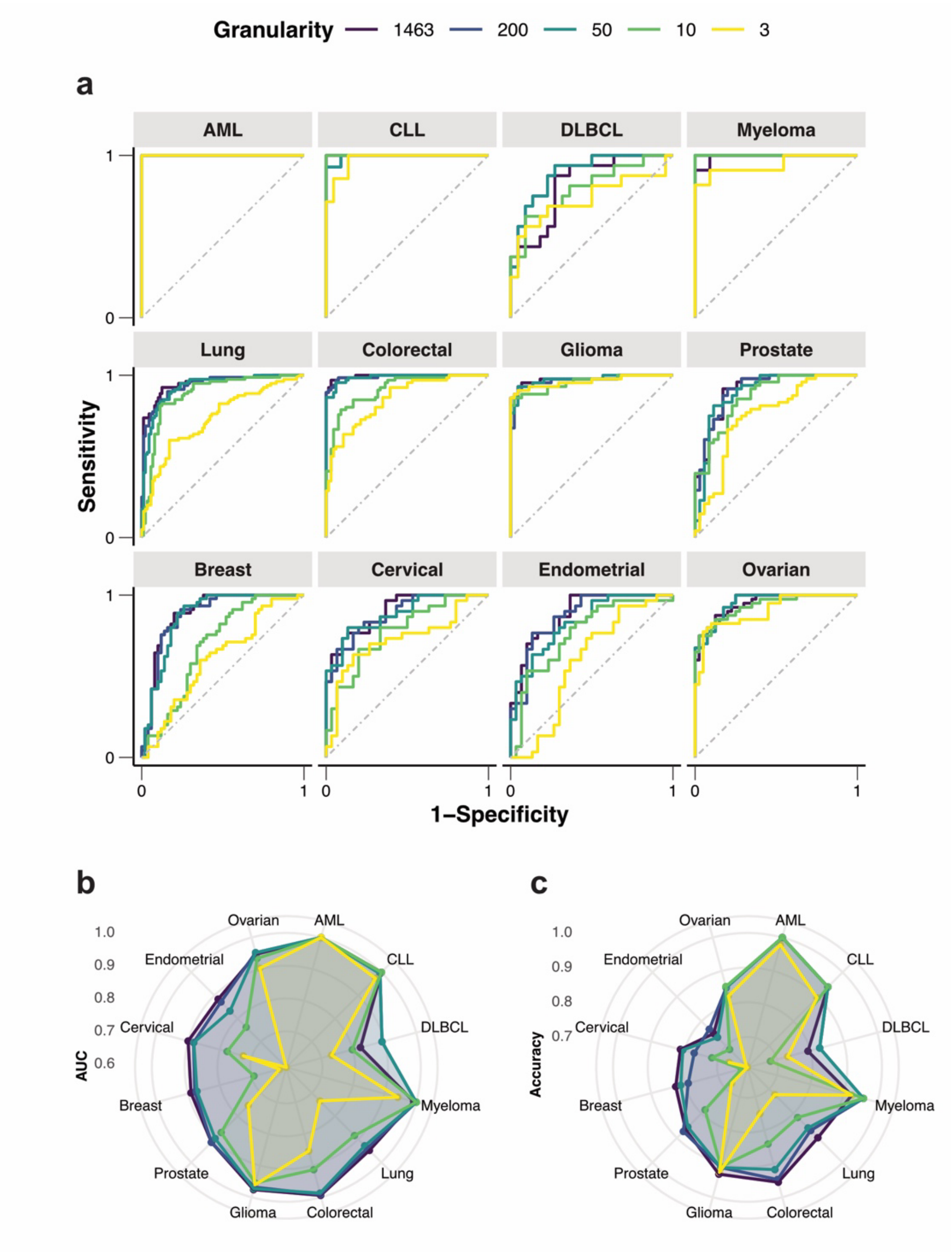

Fig. S4 | Granularity analysis for the cancer classification models. Models to classify each of the cancers individually were built using a different number of input proteins: 1463, 200, 50, 10 or 3, respectively. First, the models were run using all proteins, and subsequently, the resulting protein importance ranking was used to select the top proteins used as input for the additional models (either 3, 10, 50 or 200). Note that the total number of proteins with a positive protein ranking range between 9 for AML and 473 for colorectal cancer, resulting in identical input list of proteins for some of the granularity models for the cancers with few positive proteins. **a,** ROC curves summarizing the performance of the models with different granularity for each of the cancers. **b,** Radar plot summarizing the AUC scores for all models. **c,** Radar plot summarizing

the accuracy scores for all models. Additional performance metrics are provided for all models in **Suppl. data 4**. Source data are provided as a Source Data file. AML: acute myeloid leukemia, CLL: chronic lymphocytic leukemia, DLBCL: diffuse large B-cell lymphoma.

Figure S5

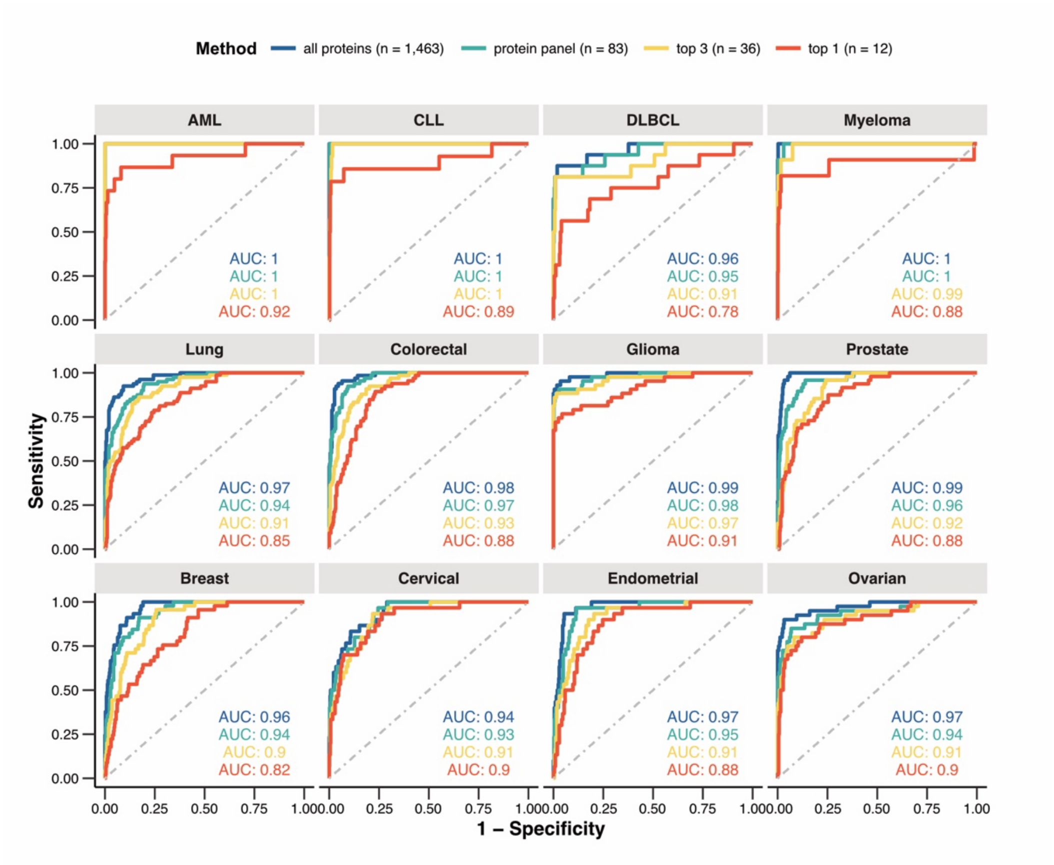

Fig. S5 | Multiclassification of the 12 cancer types using different sets of proteins. A model to classify all cancers was built using i) all proteins (n = 1463), ii) the selected protein panel (n = 83), and only the top three (n = 36) or top one (n = 12) most important proteins for each cancer. The ROC curves and corresponding AUC scores are shown for each of the cancers in each of the models. Additional performance metrics are provided for all models in **Suppl. data 6**. Source data are provided as a Source Data file. AML: acute myeloid leukemia, CLL: chronic lymphocytic leukemia, DLBCL: diffuse large B-cell lymphoma.

Figure S6

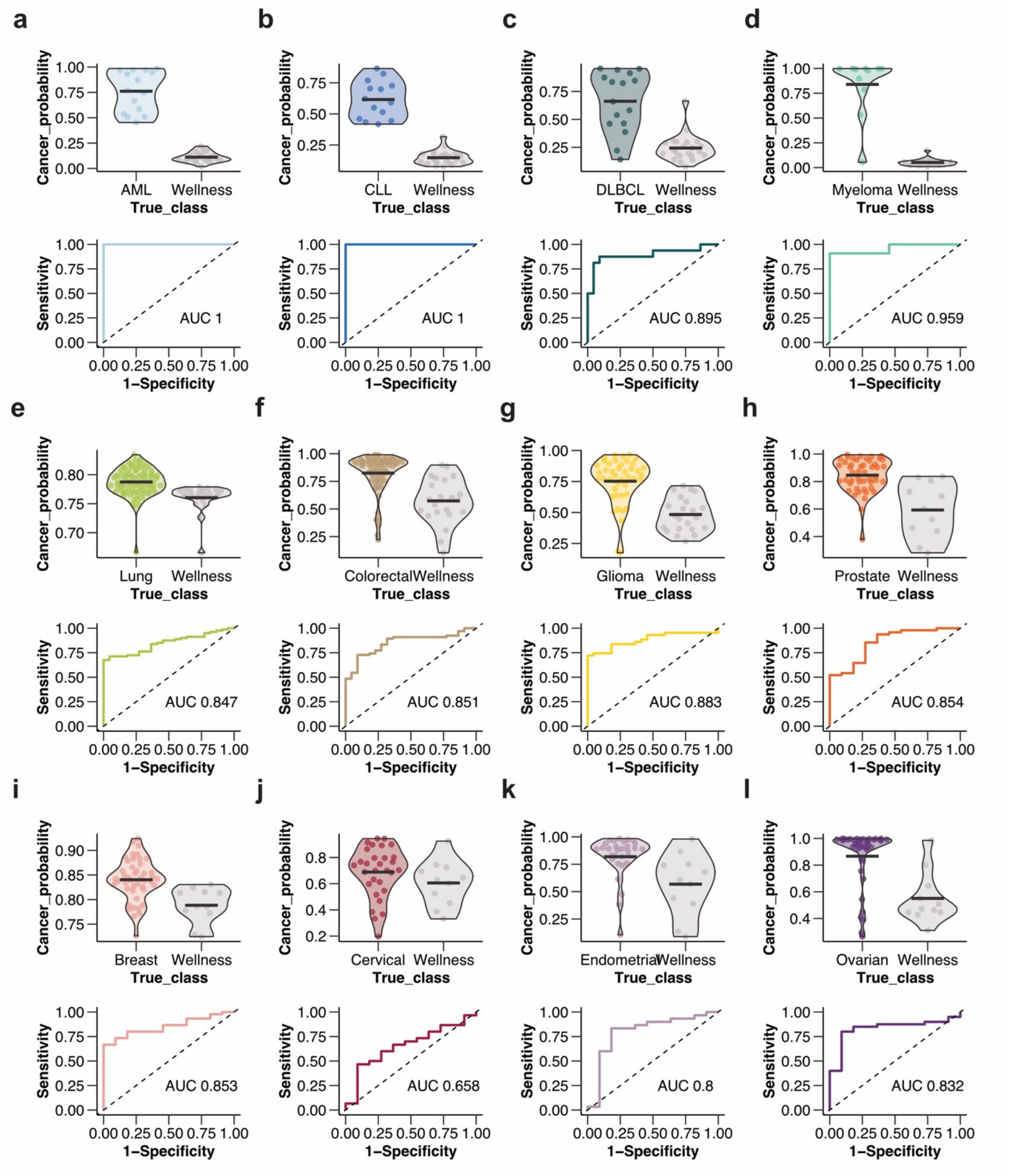

Fig. S6 | Evaluation of the prediction models built for the classification of cancer samples applied to the healthy cohort. The cancer probability for samples in the test set (top) and the corresponding ROC curves (bottom) are shown for **a,** AML, **b,** CLL, **c,** DLBCL, **d,** myeloma, **e,** lung, **f,** colorectal, **g,** glioma, **h,** prostate, **i,** breast, **j,** cervical, **k,** endometrial and **l,** ovarian cancer. The prediction models were based on

a selection of 3-18 proteins from the panel of 83 proteins for each cancer, respectively. Additional performance metrics are provided for all models in **Suppl. data 7**. Source data are provided as a Source Data file. AML: acute myeloid leukemia, CLL: chronic lymphocytic leukemia, DLBCL: diffuse large B-cell lymphoma.

Figure S7

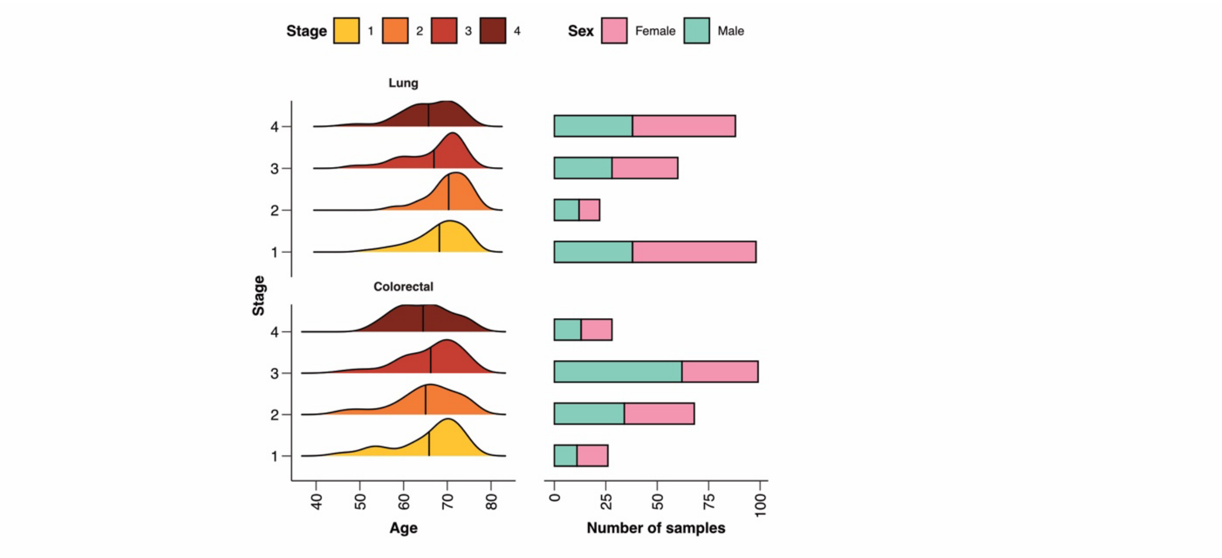

Fig. S7 | Stage-dependent analysis of lung and colorectal cancer. Age distribution and number of patients across the different stages for lung and colorectal cancer. Source data are provided as a Source Data file.
