# Supplementary material for: Next generation pan-cancer blood proteome profiling using proximity extension assay
Source: Nat Commun. 2023 Jul 18;14:4308. doi: 10.1038/s41467-023-39765-y (PMC10354027; doi:10.1038/s41467-023-39765-y)
Supplement: Supplementary file 2 — Description of Additional Supplementary Files [file 41467_2023_39765_MOESM2_ESM.pdf]

Description of Additional Supplementary Files

Supplementary Data 1 : Cohort overview. Summary of the clinical cohorts (U-CAN and Wellness), including age distribution, sex, and cancer stage and grade when applicable.

Supplementary Data 2 : Patient metadata. Summary of the clinical metadata available for the pan-cancer cohort, including cancer diagnosis, age at diagnosis, sex, and cancer stage or grade.

Supplementary Data 3 : Prediction models. Protein importance scores resulting from the multivariate prediction models for the 12 cancer types.

Supplementary Data 4 : Granular model performance. Performance metrics from the classification models for each of the 12 cancer types using a different number of input proteins (1463, 200, 50, 10 or 3). The metrics are estimated on the test set, and include AUC, sensitivity, specificity, positive predictive value (PPV), negative predictive value (NPV), precision, recall and F1.

Supplementary Data 5 : Protein panel. List of the 83 targets included in the pan-cancer protein panel including results from the disease prediction models (Protein importance) and differential expression (NPX difference between the specific cancer and other cancers and adjusted p-value). P-values are calculated using a two-sided t-test, with Benjamini-Hochberg multiple hypothesis correction.

Supplementary Data 6 : Multiclassification performance. Performance metrics from the multiclass classification models using all proteins, the panel proteins (n= 83), top 3 (n = 36) and top 1 (n= 12) proteins per cancer. The metrics are estimated on the test set, and include AUC, sensitivity, specificity, positive predictive value (PPV), negative predictive value (NPV), precision, recall and F1.

Supplementary Data 7 : Wellness models performance. Performance metrics from the models classifying each cancer from healthy patients using cancer-associated proteins. The metrics are estimated on the test set, and include AUC, sensitivity, specificity, positive predictive value (PPV), negative predictive value (NPV), precision, recall and F1.
