# Supplementary material for: Next generation pan-cancer blood proteome profiling using proximity extension assay
Source: Nat Commun. 2023 Jul 18;14:4308. doi: 10.1038/s41467-023-39765-y (PMC10354027; doi:10.1038/s41467-023-39765-y)
Supplement: Supplementary file 10 — Reporting Summary [file 41467_2023_39765_MOESM10_ESM.pdf]

Corresponding author(s): Mathias Uhlén

Last updated by author(s): Jun 22, 2023

Reporting Summary

Nature Portfolio wishes to improve the reproducibility of the work that we publish. This form provides structure for consistency and transparency in reporting. For further information on Nature Portfolio policies, see our Editorial Policies and the Editorial Policy Checklist.

Statistics

For all statistical analyses, confirm that the following items are present in the figure legend, table legend, main text, or Methods section.

n/a	Confirmed	
☐	☒	The exact sample size (*n*) for each experimental group/condition, given as a discrete number and unit of measurement
☐	☒	A statement on whether measurements were taken from distinct samples or whether the same sample was measured repeatedly
☐	☒	The statistical test(s) used AND whether they are one- or two-sided<br>*Only common tests should be described solely by name; describe more complex techniques in the Methods section.*
☐	☒	A description of all covariates tested
☐	☒	A description of any assumptions or corrections, such as tests of normality and adjustment for multiple comparisons
☐	☒	A full description of the statistical parameters including central tendency (e.g. means) or other basic estimates (e.g. regression coefficient) AND variation (e.g. standard deviation) or associated estimates of uncertainty (e.g. confidence intervals)
☐	☒	For null hypothesis testing, the test statistic (e.g. *F*, *t*, *r*) with confidence intervals, effect sizes, degrees of freedom and *P* value noted<br>*Give P values as exact values whenever suitable.*
☒	☐	For Bayesian analysis, information on the choice of priors and Markov chain Monte Carlo settings
☒	☐	For hierarchical and complex designs, identification of the appropriate level for tests and full reporting of outcomes
☒	☐	Estimates of effect sizes (e.g. Cohen's *d*, Pearson's *r*), indicating how they were calculated

Our web collection on statistics for biologists contains articles on many of the points above.

Software and code

Policy information about availability of computer code

Data collection	R project for statistical computation (version 4.0.3)
Data analysis	R project for statistical computation (version 4.0.3) ; packages: caret (version 6.0.90), clusterProfiler (version 3.18.1), ggplot2 (version 3.3.5), ggbeeswarm (version 0.6.0), ggpubr (version 0.5.0) , ggraph (version 2.0.5), ggrepel (version 0.9.1), ggridges (version 0.5.3), ggplotify (version 0.1.0), igraph (version 1.2.6), limma (version 3.46.0), multiROC (version 1.1.1), pheatmap (version 1.0.12), patchwork (version 1.1.1), pROC (version 1.18.0), tidygraph (version 1.2.0), tidyverse (version 1.3.2) and UpSetR (version 1.4.0).<br><br>All software and code is freely available at https://github.com/buenoalvezm/Pan-cancer-profiling (https://doi.org/10.5281/zenodo.7993286).

For manuscripts utilizing custom algorithms or software that are central to the research but not yet described in published literature, software must be made available to editors and reviewers. We strongly encourage code deposition in a community repository (e.g. GitHub). See the Nature Portfolio guidelines for submitting code & software for further information.

Data

Policy information about availability of data

All manuscripts must include a data availability statement. This statement should provide the following information, where applicable:

- Accession codes, unique identifiers, or web links for publicly available datasets
- A description of any restrictions on data availability
- For clinical datasets or third party data, please ensure that the statement adheres to our policy

The normalized U-CAN proteomics data generated in this study have been deposited in the BioStudies database under accession code S-BSST935 [https://www.ebi.ac.uk/biostudies/studies/S-BSST935], as well as on the Human Protein Atlas data publication page [https://www.proteinatlas.org/about/publicationdata]. All proteins are also visualized on the individual protein summary pages of the Human Disease Blood Atlas.

For the Wellness healthy cohort, the Olink Explore participant-level datasets have been deposited with the Swedish National Data Service [https://snd.gu.se/sv/catalogue/study/preview/88efa94d-39b3-4a50-8b3b-87b1abedefd4], and the data have been previously published. Due to patient consent and confidentiality agreements, the datasets can be made available only for validation purposes by contacting snd@snd.gu.se. Data access will be evaluated according to Swedish legislation. Data access for research related questions in the S3WP program can be made available by contacting the corresponding author.

Source data are provided with this paper.

Human research participants

Policy information about studies involving human research participants and Sex and Gender in Research.

Reporting on sex and gender	The biological sex of the research participants was documented as part of the sample collection process. Any self-reported gender was not documented.<br><br>This study has a tumor-centric approach where the properties of the different tumor types themselves are the main focus, rather than differences between the sexes. Thus, for those forms of cancer which both sexes can suffer, comparisons where made across the whole set of cancer forms without specific regards to sex.<br><br>However, for certain sex-specific forms of cancer, naturally only one sex was included in the analysis. Comparisons of these sex-specific cancers to other forms of cancer were limited to only include comparisons with the similar sex of other cancer forms.
Population characteristics	The U-CAN cancer cohort is a population-based cohort that recruits adult (18+) patients that seek treatment and care for cancer within the Uppsala health-care region. This means that the cohort resembles that of the general Swedish population within the hospital's uptake area. No selection based on sex, age, race or ethnicity is performed during recruitment to the U-CAN cohort.<br><br>There were no specific inclusion or exclusion criteria based on age, gender or ethnicity during the patient selection process. However, during patient selection, we tried to spread out the different cancer stages to have a good ratio of high and low stages represented for each cancer in the study.<br><br>All samples used for this study were collected around the time of diagnosis, meaning that the patients were treatment-naive at the time of sampling (to the best of our available knowledge).<br><br>The U-CAN cohort had an age range of 30 - 85, with an average of 63.30, a total of 890 female patients and 587 male patients. Details for each of the cancers are provided in Supplementary data 1 (on the group level) and Supplementary data 2 (on the individual level).<br><br>The Wellness cohort had an age range of 50 - 65, with an average of 57.09, a total of 35 female individuals and 39 male individuals.
Recruitment	Rectuitment to U-CAN is performed within the gerneral health care system within the Uppsala health-care region. Participation in U-CAN is voluntary and patients can choose not to participate. This could possibly create a bias towards only including patients that are fit and capable enough to make this decision. This could impact the overall collection/recruitment of patients into the U-CAN cohort to include fewer older patients and fewer advanced cancer stages overall, compared to the general population. However, for this study we tried during the patient selection process to spread out the different stages evenly based on the material available to us, and therefore there should be no general bias towards only having earlier stages of cancers represented in this study. All participants provided written informed consent, and there was no compensation.
Ethics oversight	The research complies with all relevant ethical regulations. The pan-cancer study was approved by the Swedish Ethical Review Authority (EPM dnr 2019-00222). The research was in line with donor consents in U-CAN (28631533, EPN Uppsala 2010-198 with amendments). The Wellness healthy cohort study was approved by the Ethical Review Board of Goteborg, Sweden (registration number 407-15), and all participants provided written informed consent. The study protocol conforms to the ethical guidelines of the 1975 Declaration of Helsinki.

Note that full information on the approval of the study protocol must also be provided in the manuscript.

Field-specific reporting

Please select the one below that is the best fit for your research. If you are not sure, read the appropriate sections before making your selection.

☒ Life sciences ☐ Behavioural & social sciences ☐ Ecological, evolutionary & environmental sciences

For a reference copy of the document with all sections, see nature.com/documents/nr-reporting-summary-flat.pdf

Life sciences study design

All studies must disclose on these points even when the disclosure is negative.

Sample size	We strived towards having as many samples as possible per diagnostic area while also having as many different diagnostic areas as possible represented.<br><br>For many common forms of cancer (e.g. colorectal-, lung-, prostate-, breast cancer) we included proportionately more cases due to the overall higher frequency of these cancer forms, and because there were enough available samples to reach >100 patients for each cancer form. However, in certain uncommon forms of cancer the low number of available samples limited the study in terms of acquiring at least 100 samples, which is why a threshold of availability of at least 50 available samples per diagnosis was set as necessary for a certain diagnosis to be included in the study.<br><br>Given the large number of samples in the study overall and the large number of analytes that were assayed, we expect that the power and stringency of the statistical models performing the many cross-comparisons between all the different diagnoses types would provide enough statistical power/certainty to eliminate most of the potentially confounding factors.
Data exclusions	In the pre-analytical phase, patients were excluded from the study if they had any other known current cancer or previous form of cancer within the last five years. Samples were likewise excluded if the arm-to-freezer time when collecting the blood samples exceeded 360 minutes.
Replication	The study employed Olink Explore technology to measure proteins in plasma. In this platform 3 assays are run in 4 different panels, which allows to ensure the reliability and reproducibility of the findings. Statistical methods were used to assess the reproducibility of the results.
Randomization	In the pre-analytical phase U-CAN samples from different forms of cancer were randomized onto 96-well plates prior to analysis, in order to create a mixture of represented diagnoses, participant ages, and years of sample collection. After analysis of the samples, the acquired data was grouped according to the patient's cancer diagnoses.
Blinding	Investigators were not blinded to clinical data during the process of selecting patients to be included in the study. During the data-acquisition and QC-steps, operators were blinded to the contents of the plates. After obtaining data, the connection between the obtained data and individual's diagnoses were re-established in order to perform the grouping into different diagnostic sets. Blinding was applied to data analysis when possible, for example when testing the machine learning models on unseen data.

Reporting for specific materials, systems and methods

We require information from authors about some types of materials, experimental systems and methods used in many studies. Here, indicate whether each material, system or method listed is relevant to your study. If you are not sure if a list item applies to your research, read the appropriate section before selecting a response.

Materials & experimental systems

n/a	Involved in the study	
☐	☒	Antibodies
☒	☐	Eukaryotic cell lines
☒	☐	Palaeontology and archaeology
☒	☐	Animals and other organisms
☐	☒	Clinical data
☒	☐	Dual use research of concern

Methods

n/a	Involved in the study	
☒	☐	ChIP-seq
☒	☐	Flow cytometry
☒	☐	MRI-based neuroimaging

Antibodies

Antibodies used	All antibodies used in the study are publicly available through standarized kits provided by Olink Proteomics (www.olink.com). The Olink Explore 1536 platform includes four different panels: the Olink Explore 384 Cardiometabolic Reagent Kit (Panel lot number: B04413, Product number: 97700/97300), the Olink Explore 384 Inflammation Reagent Kit (Panel lot number: B04411, Product number: 97500/97100)), the Olink Explore 384 Oncology Reagent Kit (Panel lot number: B04412, Product number: 97600/97200)), and the Olink Explore 384 Neurology Reagent Kit (Panel lot number: B04414, Product number: 97800/97400).

Validation	The analytical performance of the antibodies have been validated for sensitivity, dynamic range, specifciity, precision and scalability, and the results are summarised in the Data Validation documents for each panel, which can be downloaded from the validation data website (www.olink.com/data-you-can-trust/validation).

Clinical data

Policy information about clinical studies

All manuscripts should comply with the ICMJE guidelines for publication of clinical research and a completed CONSORT checklist must be included with all submissions.

Clinical trial registration	Not applicable.
Study protocol	Not applicable.
Data collection	Retrospective clinical data was obtained from hospital/patient records as part of the U-CAN project's ongoing sample and data collection process. Patients in this study were included in U-CAN between 2010 and 2018 at the Akademiska Hospital in Uppsala, Sweden. Samples were obtained at the time of diagnosis and stored at Uppsala biobank as part of the U-CAN sample collection.
Outcomes	Cancer diagnosis was defined as the primary outcome for the study.
